# Brands, networks, communities: How brand names are wired in the mind

**DOI:** 10.1371/journal.pone.0273192

**Published:** 2022-08-25

**Authors:** László Kovács, András Bóta, László Hajdu, Miklós Krész

**Affiliations:** 1 Savaria Department of Business Administration, Faculty of Social Sciences, E¨otv¨os Lor´and University, Szombathely, Hungary; 2 Department of Computer Science, Electrical and Space Engineering, Embedded Intelligent Systems Lab, Lule˚a University of Technology, Lule˚a, Sweden; 3 Innorenew CoE, Izola, Slovenia; 4 Faculty of Mathematics, Natural Sciences and Information Technologies, University of Primorska, Koper, Slovenia; 5 Andrej Maruˇsiˆc Institute, University of Primorska, Koper, Slovenia; 6 Gyula Juh´asz Faculty of Education, University of Szeged, Szeged, Hungary; University of Sao Paulo, BRAZIL

## Abstract

Brands can be defined as psychological constructs residing in our minds. By analyzing brand associations, we can study the mental constructs around them. In this paper, we study brands as parts of an associative network based on a word association database. We explore the communities–closely-knit groups in the mind–around brand names in this structure using two community detection algorithms in the Hungarian word association database ConnectYourMind. We identify brand names inside the communities of a word association network and explain why these brand names are part of the community. Several detected communities contain brand names from the same product category, and the words in these categories were connected either to brands in the category or to words describing the product category. Based on our findings, we describe the mental position of brand names. We show that brand knowledge, product knowledge and real word knowledge interact with each other. We also show how the meaning of a product category arises and how this meaning is related to brand meaning. Our results suggest that words sharing the same community with brand names can be used in brand communication and brand positioning.

## Introduction

Brands can be seen as companies’ most valuable assets. The literature describes brands in different contexts, e.g. distinguishing symbols or entities influencing consumer decisions [1–4]. A recent overview of the different approaches to brands is provided by Esch [5], showing how the definition of brand evolved over the years, how the perception of brands changed and why different brand viewpoints are possible.

One possible research direction interprets brands as entities of the mind (cf. e.g. Franzen and Bouwman in [6]). Brands, in this regard, can be seen as psychological constructs, exerting their effect only in the mind of the consumer: ‘a brand is something that resides in the minds of consumers’ [7].

Brands occupy a specific position in the mind. This mental position can be determined by the words (and concepts) associated with brands [6, 7]. These associated words not only define a position for the brand in the mind, but they also create the image and the meaning of the brand. Keller sees brand associations as an important part of brand image and thus also as part of brand knowledge [8]. According to him, type, favorability, strength and uniqueness of brand associations all contribute to brand image. Later, Keller points out in [9] that brand image and brand meaning are closely related, and associations are paramount for forming brand meaning and brand image. Franzen and Bouwman also show that brands evoke and are connected to all kinds of associations in our minds [6]. These associations can be words or other sensory information, including smells, sounds, tastes and images, and these associations form a brand’s meaning. Brand meaning can originate from the product category, product attributes, situations in which the brand is used and symbolic meanings [6]. Batey sees associations as a source of brand meaning; he identifies core associations around brands, which create the brand’s primary meaning [10].

Both Aaker [1] and Keller [8] agree that brand associations are an important part of brand image, thus also part of brand equity. Brand association maps drawn from brand associations can be used for 1) brand audit, 2) brand positioning, 3) portfolio management, 4) crisis management and 5) communication management [11: 97–94] (cf. also [12, 13]). Gong et al. [14] show that associations retrieved from social media posts can show a brand’s competitive advantage.

As Dzyabura and Peres [15] point out, associations–in their case, visual associations–can be used to find differences and similarities in the perception of brands, suggesting, for example, which brands could cooperate with each other. Wang et al. [16] emphasize that associations help to 1) process information connected to brands and 2) differentiate from each other and assist in brand positioning. In addition, Batey [10] points out that brand associations help to define the meaning of a brand. All of this research implies that brands’ mental representations (based on associations) describe how consumers perceive a given brand and are reliable sources for brand management.

As we see, brand meaning can be defined through the associations connected to a brand. For this reason, the associations that brand names evoke in our minds are important brand assets. But equally important are the words which evoke the brands–the cues from which a brand name comes to our minds [6]. Since these structures are less studied, analyzing which words evoke brand names and what structures brand names are part of in the mind are important and novel research directions.

Word association databases provide an opportunity for analyzing cues that evoke brand names. The data in these databases are word association data, where a cue word is presented, and respondents have to name the first word that comes to their mind when seeing the cue word. Brand names regularly occur in these databases, and they enable us to gain an insight into the mental representations and connections of brand names.

In our paper, we analyze one such database with methods from network science. We examine the connections around brands; we search for communities–closely-knit word groups–of the database with two different detection algorithms.

In the first part of the paper, we describe associations. We then show why association databases can be seen as networks and how networks and communities can help us to better understand the structures of word association data. Finally, we show that brands in the mind can be analyzed as parts of the mental network.

In the second part of the paper, we analyze the brand-related communities of a Hungarian word association database and show communities in which brand names appear. For finding communities, we use the hub percolation method [17, 18], the modularity maximization algorithm of Blondel [19], and the LEMON algorithm [20]. Our data indicate that brand names are present in the mind’s communities and that not only one but several brand names can be part of a given community. We show that communities contain brand names from the same product category.

Based on the results and previous research, we define how brand names are stored in the mind’s communities, and we show that these word communities share brand and product characteristics.

## Literature review

### Associations

Words–or more precisely, lexical units–in the mind carry pieces of information; information about a word’s frequency, meaning, morphology, phonology and syntax is stored together with pragmatic, stylistic, graphemic and encyclopedic knowledge [21, 22].

Words stored in the mind can be researched by collecting and analyzing word association data. In word association experiments, a cue word is presented to the participants, and they must write down or name the first word that comes to their minds. The method has been known since Galton [23, 24] and used since then in psychological and psycholinguistic experiments.

Word associations can be collected in several ways; participants can name a single association or multiple associations. Associations can be restricted where some characteristics of the allowed answers are determined (e.g. the word class of the response). In some experimental settings, participants are asked to name all of the associations that come to mind (i.e. continuous associations). The used methods, their variants and early results of word association research are described by Cramer [25].

Word association data is never the data of one individual; data is collected from hundreds or thousands of participants. Thus, the database represents not just the connections of one individual; rather, it represents an aggregate of all the connections we can build between words. Word association research has created several word association databases [26–30].

Since the 2000s, the method has experienced a renaissance; new, large-scale data is available, and methods from other disciplines–above all network science–can be used to analyze and describe the characteristics of the connections in the mind [31–33]. One database is the Small World of Words [34] projects, collecting word associations online. However, it is unknown exactly how many brand names are part of the database; a random search of the available online data suggests that at least some car brand names are part of the database (for some results, see [35, 36]). Another somewhat older data collection is from Human Brain Cloud [37], which has collected data since 2007. It also has brand names (e.g. on 10.02.2022, the stats page’s strongest association is Rubik’s → cube), but since it is currently not searchable, it is hard to know exactly which brand names are part of the database (for some results connected to the database, see [38]).

All in all, word association networks can be used for the description of several cognitive tasks, for example, predicting lexical norms [39], memory recall tasks [40], change of associative networks across the life span [35, 41] or description of the mental lexicon’s multilayer characteristics [36].

### Networks and communities

The concept of graphs (or networks) has been studied extensively, first as a branch of discrete mathematics. The usefulness and expressivity of the concept became increasingly apparent as other fields started adopting it into their methodology, including sociology, biology, economics and linguistics [42]. Networks are defined as a set of nodes representing items, connected by a set of links or edges, denoting some relationship between the nodes. Links between the network nodes might have designated directions signifying that the relationship is not symmetric. In this case, we talk about directed networks. In weighted networks, we can also assign weights to the links. Networks can be used to represent a large variety of real-life concepts. Social networks define relationships between people based, for example, on friendship, common interest, or work [43, 44], while economic networks may represent connections between companies or banks [45–47].

Networks also provide a natural way to describe word associations; words are connected when one word–the cue–primes another [31, 32]. These networks are both directed and weighted. Direction arises in the cue answer direction, and weight is established based on how many respondents gave the same response to the cue. For example, when a person answers wave to the cue sea, the directed edge sea →wave is created. The weight assigned to the connection corresponds to the number of people giving the same response.

Networks in real life share several common characteristics. One of these is community structure; a network’s nodes can be grouped into sets so that the nodes inside the sets are densely connected, while the links between the sets are relatively sparse [48, 49].

In social networks, this phenomenon corresponds to the tendency of people to form groups according to common interests and occupations. Word association networks also have a distinct community structure. It has been shown that the communities have an overlapping character in word association networks [50], and word communities form groups according to common semantic or grammatical information [50–52] or phonetic similarities [cf. 52, 53]. Communities can also help analyze label-homogeneous communities of the mental lexicon [54] and provide new insights into the study of semantic frames as communities [55].

Community detection, the identification of communities in a network, is a very popular subfield of network science, and a great many algorithms have been proposed to solve this task. The majority of the literature defines communities in undirected, unweighted networks and does not allow overlaps between the identified groups. The most notable approaches include modularity maximization methods [19, 42], stochastic blockmodels [56, 57] and hierarchical clustering [58, 59]. A large fraction of the community detection methods considers overlaps between communities to be a natural property of such groups, giving rise to the subfield of overlapping community detection. Methods include clique-based approaches [17, 18, 50, 58, 59] and label propagation [60], or they can extend non-overlapping methodology to allow overlaps [61]. However, only a smaller set of algorithms can handle weighted or directed networks [18, 50].

### Brands and networks in the mind

The idea of mental networks has a long history in psychology and linguistics [62, 63]. In these early publications, human memory is described as a network where nodes and connections exist.

Similarly, it has been assumed since the 1990s that brand names are also part of these networks; they are represented by nodes in memory and connected via different types of connections to each other, brand and product characteristics and other words [64]. Brands as part of (mental) networks became the focus of marketing research when Aaker [65], Keller [8], Krishnan [64] and Franzen [66] showed that brands are part of an associative network, and Esch [67] described the identified structures as schemas.

Early research only formulated assumptions on how the network structures around brands are organized–empirical data was missing [68]. Research was focused on theoretical questions or relied on data collected from a small number of subjects, which was used merely as an example [69].

There are several possible methods to analyze and create these brand-related networks. One of the earliest empirical analyses of networked structures around brands is from Henderson, Iacobucci and Bobby [68, 70], who emphasize that methods used in (social) network research can also be applied to brand associations. Using empirical data connected to car brands, the authors show which network measures can be useful for analyzing different brand-related contexts, like brand dilution and brand confusion.

The most often used method is creating Brand Association Maps [13, 69, 71], from which different metrics can be created, for example, the brand association network value (BANV), which considers both the network structure of brand associations and the associations’ favorability [72]. Empirical results show that the created associative networks around brands are also related to brand knowledge and experiences with brands [13], and they are culture-dependent, as shown by the example of Corona Extra, a Mexican beer brand [69, 71].

### Brand meaning vs. product meaning

#### Brand meaning

Assuming that brand names are stored like lexical units, phonological and graphemic information and meaning are also stored with brand names. However, the meaning of a brand name arises differently than a word’s meaning. A brand itself can be seen as ‘a cluster of meanings’ [10: 6], where a brand is ‘the consumer perception and interpretation of a cluster of associated attributes, benefits and values’ [10: 6].

Based on existing research [73, 74], we assume that a brand’s meaning arises at least through associative structures in the following contexts:

- product category
- country-of-origin
- logo
- colors
- slogan
- experiences with the brand
- experiences of others with the brand (see also [1, 6, 7].

Product category is the most important information related to a brand [72], and country-of-origin can facilitate the acceptance of a given brand [75]. Brand knowledge is also connected to cultural knowledge and awareness, for example, whether the brand–or the brand’s country-of-origin–is liked or disliked in a given culture [69, 76].

Logos themselves–their symmetry, descriptiveness–also bear a meaning that is transferred to the brand [77, 78], and logo colors can also contribute to the perceived characteristics of a brand [79].

Brand users have their own experiences with the brand [80]. However, personal experiences are stored apart from the experiences other brand users report (cf. Olson, Fazio and Han [81]; Ziegler [82]). Both the users’ experiences and the experiences of others contribute to the meaning of a brand (cf. in detail Jaki´c et al. [83]).

As a consequence, brand names are different from words; as multimodal units, they contain multisensory information about the brand’s logo and colors as well as experiences and attitudes connected to the brand.

#### Product meaning

In her book [84], Kastens assumes that information about brands is stored in a network-like structure in the mind called schema (cf. Esch [67, 85]), which is also connected to the schema of the product itself. The two–brand and product–interplay with each other, where product-specific information is stored at the product schema level (e.g. a car has wheels) and brand-specific information at the brand level (e.g. the car’s logo).

**Fig 1:**
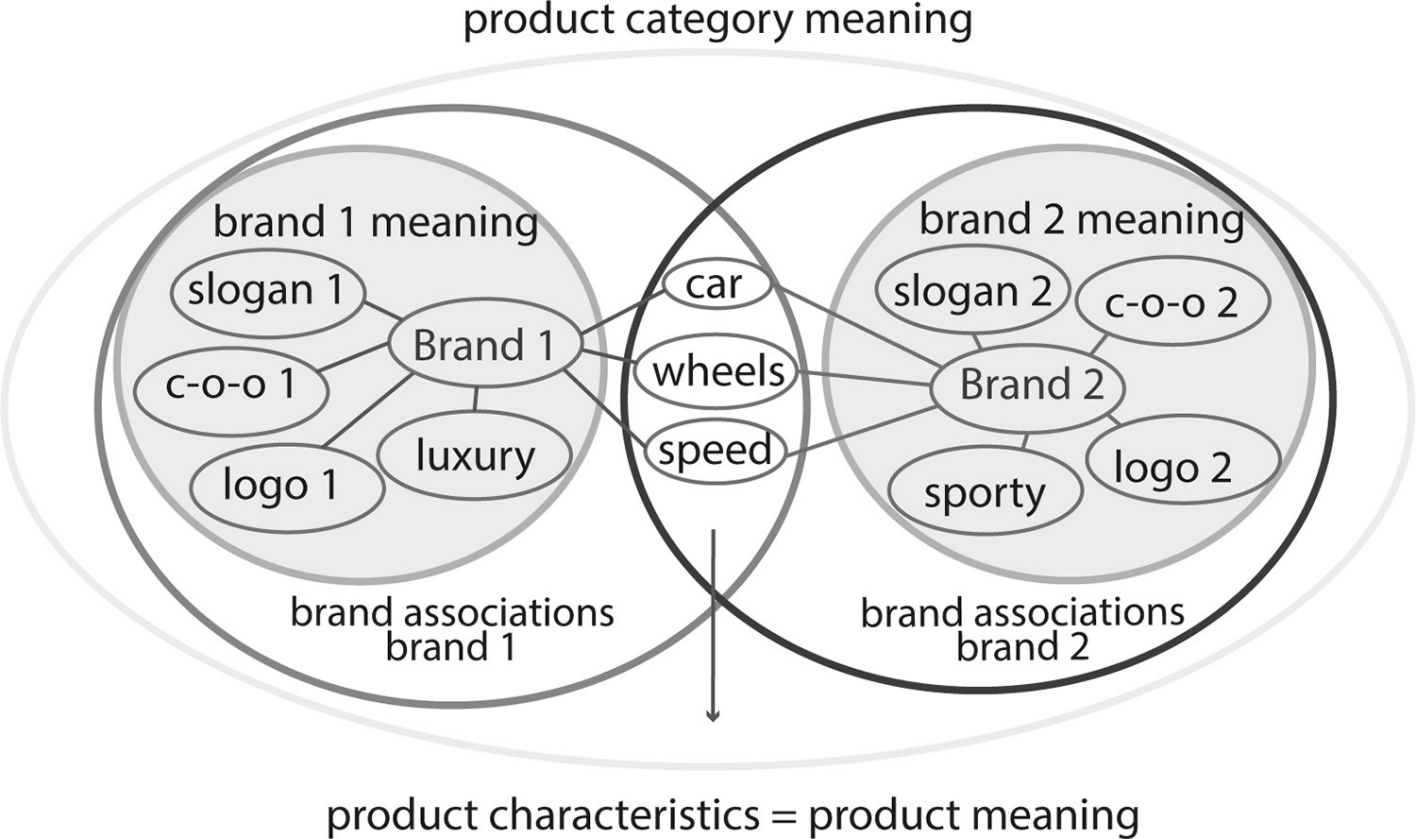
The interaction of brand associations, brand meaning, product characteristics and product category meaning in the example of two automotive brands.

In Fig 1, we show how the meaning of a product category arises; it is compiled from the brand meanings of brands belonging to the product category. It must be noted that brands share associations, which describe the product itself (e.g. each product of the product category ‘car’ shares common product characteristics). As seen in the figure, brand meaning arises from the associative structure around brands, and brands of a product category share product characteristics (cf. Kastens, product and brand-specific information [84]).

Product category meaning includes brand meaning and product meaning. Brand meaning and product meaning complement each other; while brand meaning is unique, it must include product meaning, and each brand contributes to the meaning of the product category.

Works discussed in the previous paragraphs had brands in their focus. Associations to brand names were collected, and several brand names were selected as starting points for research. However, brands’ associative structures can also be activated when the cue itself is not a brand name. Examples of this phenomenon frequently appear in word association databases [86, 87]. Here, the cue words are not brand names, and the goal is not to evoke or analyze brand names but to conduct linguistic research. Nevertheless, brand names appear in such databases, and the evoked brand names describe not only the cultural and historical context when the data was collected but may also indicate respondents’ age and gender [86, 87]. In the following, we combine word associations, brand names and communities; we analyze the communities of a Hungarian word association database to see which brand names are part of the same communities.

We seek to answer the following research questions:

RQ1) Are brand names parts of communities in the mental lexicon?
RQ2) Are overlapping or non-overlapping methods better for brand community detection?
RQ3) What is the advantage of analyzing communities around brands instead of associations connected to brands?

Based on the results, we describe how brand names are represented in the mind and show the implications of the results.

## Materials and methods

### Data

Research data used in this paper was collected from the word association database ConnectYourMind, which collected associations online between 2008 and 2014, primarily in Hungarian. During that period, approximately 182.000 associations were collected from 1035 respondents.

The data collection was a classic word association experiment online. Participants were shown cue words, and as a response, they had to write down any words or combination of words that came to their mind. In the experiment, no restrictions were given according to the responses; any word or part of a sentence (up to 255 characters) could function as a response to the cue words. Responses could be proper names or inflected forms. The language of the responses was also not restricted; thus, the website also collected data–although only several hundred words–in English, German and Italian.

The data was collected in two ways; the first 134 words appeared in a given order to all respondents. These 134 words were the initial cue words of the database [33]. After these words, words from the database were presented to respondents in a random order as cue words. Thus, responses themselves also functioned as cue words, enabling the collection of word association data for a large number of words. Brand names were not part of the original word list. However, brand names appeared as responses in the database; therefore, during the data collection, they also functioned–in several cases–as randomly presented cue words and collected some association. Brand names are part of the database both as responses (in many cases) and cue words (in a few cases).

Participants took part in the experiment voluntarily, and they could end the experiment anytime by simply navigating away from the webpage. Details of the data collection are described in more detail in [52] and exhaustively–although in Hungarian–in [33].

While the mean number of responses per user was 156, the standard deviation of this value was 155, showing that there was a considerable difference in user patience. This also means that the first cue words received more associations than the rest, and we acknowledge this as a limitation of our work.

**Fig 2:**
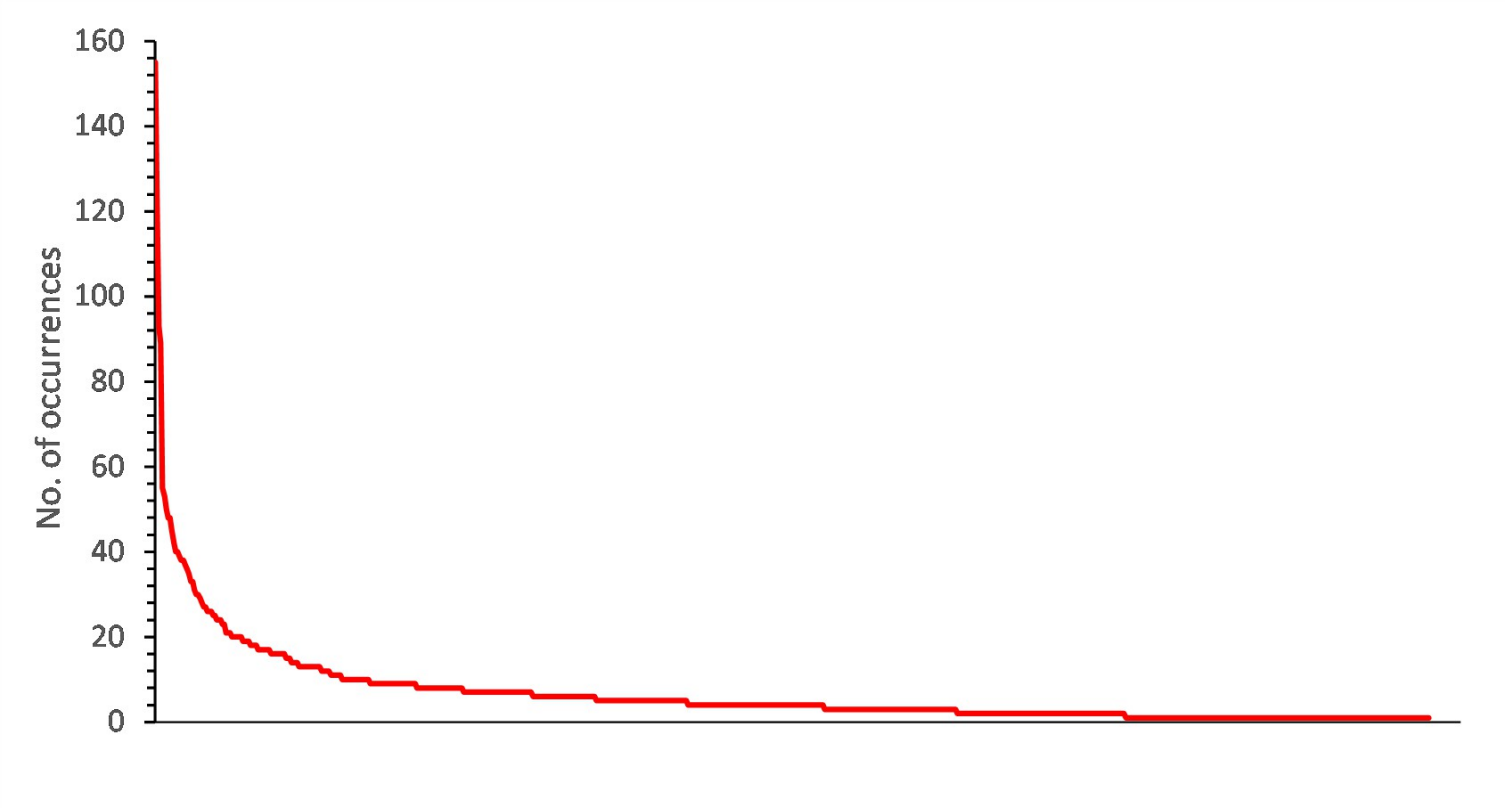
The distribution of brands. Brand names which have also a meaning in Hungarian are omitted.

The database contains 699 brands annotated by an expert. The distribution of brands inside the word associations is highly inhomogeneous, with the top 50 brands receiving 50% of the total number of associations containing brands (Fig 2.). As we can see, the distribution of the associations to brand names is a heavy-tailed distribution, as observed in other semantic and word association networks (cf. [27, 88, 89]).

### Association network

The network itself is constructed in the following way: the connections between words represent the cue word–association relationship. In the network, each word represents a node, and each given association (response) is represented as a directed, weighted edge in the form of a cue word → response. For example, to the cue word “apple”, a respondent typed in “pear”; in the database, the word “pear” was stored, and a connection between them was created: apple → pear, with a connection strength of 1. When a second person gave the same answer (again pear to apple), the strength of the connection was increased (resulting in apple → pear, connection strength of 2). In the database–due to the data collection process–the first 134 words collected more associations and enabled a deeper analysis of the associative structure for these words [33]. On the other hand, the responses functioning as cue words enabled the creation of a large database with many cue words and responses.

### Algorithms and community detection

We used community detection to create the groups of associations examined in this paper. We applied several detection algorithms to determine which one fits our specific application best. We have experimented with the following non-overlapping, overlapping and directed algorithms:

The hub percolation method [17] is a clique-based method originally defined for undirected networks as well as unweighted and weighted ones. It is a flexible method with tunable parameters that govern the properties of the resulting communities, like the number and size of the overlaps between them. The method can also take the edge weights of the network into account when constructing the communities. The hub percolation method relies on maximal cliques (fully connected subgraphs) as the building blocks of community detection. The method uses the concept of hubs (central nodes) to connect these building blocks according to a percolation rule [50] in order to create communities.

Directed hub-percolation [18], the directed version of hub percolation, uses directed cliques instead of undirected ones.

The modularity maximization method of [19] is based on one of the first and most popular metrics to measure the output quality of non-overlapping community detection methods. The modularity metric proposed by [49] compares the discovered edge distribution of the input network’s community structure with the edge distribution of the configuration model: a random network with the same degree distribution of the original, but without community structure. Blondel’s algorithm is a greedy optimization method based on modularity. Starting from a state where each node has its own community, it iteratively joins communities so that the net modularity gain is maximal. The algorithm stops if no joining operation results in an increase in modularity. We used the implementation available as part of the Gephi software package [90] to compute our results.

The Infomap algorithm [91] seeks to minimize a different cost function: the map equation. The algorithm seeks to find a compressed representation of random walks on the graph, using the intuition, that the shortest representations involve clusters of nodes as opposed to individual ones. The results can then be naturally interpreted as communities. We used the non-overlapping implementation available from [92].

Stochastic blockmodels [93] are generative models for random graphs that can be easily set up to produce graphs with community structure. Models can be defined for both overlapping and non-overlapping communities. Stochastic blockmodels can be inferred from instances of networks, and stochastic blockmodel inference (SBM) [94] became a popular approach in the previous decade. We used the overlapping implementation available at [95].

The LEMON algorithm is an overlapping detection algorithm based on local expansion via minimum one norm [20]. The method aims to extend an input seed set, following the reasoning that the seeds are initially known members of yet unknown communities, and the goal of the detection method is to find the remaining members of said communities. Since the goal of this study is to explore communities around brand names, we used a list of brand names identified by our expert as a seed set for this algorithm. We used the implementation available at [96].

We applied all of the above detection algorithms to the Hungarian word association network and measured their performance according to multiple metrics, such as conductance [97], hub-dominance [98] average transitivity [99] and non-overlapping modularity [100]. Table 1 shows the metrics and other characteristics of the community structure.

**Table 1:** Properties of the results of the community detection algorithms in the leftmost column. Modularity was only computed for Blondel’s (non-overlapping) algorithm. For directed hub-percolation, only out-neighborhood was considered when calculating these metrics. Overlapping algorithms are marked in italic text.

	Number of communities	Average community size	Conductance	Average transitivity	Hub-dominance	Modularity (non-overlapping)
Modularity maximization	692	10	0.578	0.0361	0.6951	0.1361
*Hub-percolation*	2716	31	0.9362	0.7509	0.9521	
*Directed hub-percolation*	225	1025	0.8273		0.5347	
*LEMON*	672	65	0.8033	0.1774	0.5716	
*SBM*	9	150	0.9674	0.1216	0.3471	
Infomap	1772	11	0.5539	0.0377	0.5538	0.3092

We can see that different methods capture completely different aspects of the community structure of the word association network. Modularity maximization produces low-transitivity communities organized around hubs, with relatively low modularity for the end result. As can be expected from a clique-based method, hub-percolation identifies many small, quasi-clique communities with high transitivity and hub-dominance. The directed version of the algorithm produces the opposite: few large communities. SBM inference could not find a sufficient number of communities. The LEMON algorithm had the advantage that community seed candidates could be provided as inputs of the algorithm. We used expert-annotated brand names for this purpose.

However, since our aim in this paper is to examine the association network’s communities from the perspective of brand analysis, we ultimately selected the methods that promised to provide the most meaningful results from this practical perspective. These were undirected hub-percolation, the LEMON algorithm and modularity maximization. The LEMON algorithm was especially useful in our analysis, since we are interested in communities organized around a priori known brand names and we could simply provide a list of brand names to the algorithm as input, which would then grow communities around them. Both the star-like structure of the communities identified by the modularity maximization algorithm, and the high-resolution quasi-clique communities found by undirected hub-percolation were easy to interpret.

## Results

In the first step of our analysis, we searched for communities in our word association network with the two community-finding methods defined in the previous section. In the identified communities, we searched for brand names manually. We define brand names as any name that can influence consumer decisions. We do not differentiate if the brand name is a registered trademark or not, an umbrella brand name or an individual brand name, or a product or a services brand.

### Results with the Hub percolation method

The first detection algorithm we used was the hub percolation method [17, 18]. The communities found by this method are overlapping communities, and the network is considered to be undirected–only the existence of a connection was important, without the direction of the connection. The algorithm detected 2716 communities, with 63328 words in these communities, where communities have 3 to 255 members. Communities detected with this approach are overlapping; a word may belong to multiple communities. This is in line with the observations made in [52].

Altogether 81 communities contain brand names. Large communities– 70+ words–are heterogeneous (including several topics), and if more brand names appear in one community, they are partially from different product categories.

The picture changes when the communities become less than 70 words. In this case, the communities containing a brand name tend to contain more brand names, and these brand names are clearly related to each other. Like in community nr. 111, containing 66 words, the brand names *CIB*, *OTP*, *MKB*, *CIB Bank* and *Erste* appear–all brand names of banks operating in Hungary. The same applies to community nr. 183 (54 words), containing the brands *Alcatel*, *T-mobile*, *v630i*, *Nokia*, *Sony*, *Blackberry*, *Motorola*, *LG* and *Samsung–*mobile phone brands and a service provider from the pre-smartphone era in Hungary (note: data collection took place between 2008 and 2014). Likewise, community nr. 642 (30 words) contains *Nokia*, *iPhone*, *Apple*, *Motorola*, *Samsung*, *HTC* and *Sony*. It is also interesting that if a community has one brand, it likely has another. However, some communities contain brands from different product categories; community nr. 116 (65 words) contains *Gucci*, *Mercedes*, *Bugatti* and *Audi*, of which three are car brands and one is a fashion brand. However, all can be considered brands in the luxury segment of the given product category; thus, they share the same segment, although in different product categories.

The pictures change again in communities with less than 20 members; communities containing brand names only have a single brand name. The picture from the used community detection method is not clear. Communities having between 20 and 70 members seem to be homogeneous considering brand names. Under a homogenous community, we will understand a community where the brand names are from the same, or closely related, product categories; for example, when only vehicle brand names appear in a community, we consider it as homogenous regarding brand names. Communities above this size are heterogeneous, and below this size, they contain only one brand name as a member.

### Results with the LEMON method

Using another overlapping algorithm (LEMON), all communities are guaranteed to have brand names. The communities found by the algorithm are–according to brand names–homogenous. The brand names in a given community belong to the same product category or closely related product categories; for example, communities nr. 4 and 6 consist of vehicle brands (in the case of nr. 6, 81 words, including 20 brands: *Citroën*, *Xsara*, *Honda*, *Accord*, *Yamaha*, *Toyota*, *Suzuki*, *Avensis*, *Peugeot*, *Mx-5*, *Subaru*, *Volkswagen*, *Alfa Romeo*, *Fiat*, *R5*, *GTO*, *Polo*, *Micra*, *Nissan*, *Suzuki Hayabusa*), community nr. 23 has lifestyle brands, mostly watch brands (81 words, including: *Rolex*, *Oakley*, *Fossil*, *Citizen*, *Swatch*, *Casio*, *Tag-Heuer*, *Breitling*, *Tissot*) and community nr. 280 has 40 members with the brands *Gucci*, *Dior*, *Armani* and *Prada*.

However, heterogeneous communities–brands from different product categories–were also identified, for example, community nr. 62., 81 words, 29 brands: *Bugatti*, *Lexus*, *BMW*, *M7*, *Allianz*, *Ferrari*, *Nikon*, *Reserved*, *Ralph Lauren*, *Dolce*, *Mango*, *Benz*, *Mercedes*, *Nike*, *Ford*, *Dodge*, *Viper*, *Phillip Russel*, *Oakley*, *Milka*, *T-modell*, *Cora*, *Mazda*, *Opel*, *Coca-Cola*, *Dior*, *Renault*, *Toyota*, *Gucci*, together with the words *brand*, *trademark* (védjegy) and *prestige* (presztízs), and with the expression *expensive things* (drága dolgok). It is clear that this community is centered around (expensive) brands; hence, brands of different product categories are present.

It is clearly impossible to verify one-by-one the consistency and all words of all communities; it can be said, however, that from brands’ point of view, most communities seem to be homogenous. The algorithm produces good, overlapping results; thus, due to the overlapping nature of the method, it is possible to see several community structures the brand is part of–on the other hand, the analysis is impeded exactly by the fact that several contexts have to be analyzed and minor differences must be accounted for. For example, the brand name *Citroën* is part of 24 communities, *Ferrari* of 18 communities, and *Nikon* of 31 communities. This means that based on this database, to have a clear picture of the brand *Citroën*, all words in 24 different communities should be researched, and the connections between the words should be analyzed.

### Results with the Modularity maximization method

To refine our results, we decided to use the modularity maximization algorithm of Blondel et al. [19] to see if it detects more homogeneous communities. The algorithm enables us to identify smaller (two-member) communities compared to hub percolation. It is strictly non-overlapping; however, each node can be part of only one community. We applied the modularity maximization method in two stages. In the first step, we searched for communities containing associations named by at least two participants to ensure that the found communities are not dependent on individuals. The algorithm identified 693 communities containing 8710 different words. The community sizes ranged from 2 to 290 members, in line with the observations made in [52].

Of the 692 communities, 32 contained brand names. With this method, the brand names in the communities were from the same category or product group. The communities detected with these methods were more homogeneous than the ones detected by the hub percolation, making it possible to determine the key words and ideas the community is structured around. The key words of the community were identified manually by the authors. We used the automatic detection method–manual analysis in line with Brookes and McEnery [101].

For example, in the community ‘credit and banking’, the appearing brand names are all banks (11 brand names from 84 words); in the community ‘shopping’, they are supermarkets, discount supermarkets and local chains (15 from 148 words); in the community ‘time’ (54 words), two watch brands, *Rolex* and *Swatch* appear; and in the community ‘fashion’ (83 words), we found the fashion brands *Gucci*, *Armani*, *DG*, and *Mayo Chix* along with *A´rka´d* (a shopping center), *Takko* (a textile discount store) and *L’Or´eal*. In this community, we found the city names *Paris* and *Milan* too. In the community ‘reading and news’, brand names of newspapers, magazines and television channels appear (31 from 145), and names of writers *(Stephen King*, *Robin Cook)* and fictional characters *(Bridget Jones)* are also present.

All communities found with the modularity maximization method were homogeneous, with one exception: a community centered around fruit names and colors (242 words). It contained the name *Ferrari*, *Greenpace*, *Fradi* (a Hungarian soccer team with distinctive green color), *Jonathan* (an apple name), *Snow White* and the *Smurfs*, with their Hungarian name *Hupik´ek T¨orpik´ek* (‘blue smurfs’). The last two are brand names usable for merchandising and licensing (cf. Lind [102], Simon [103]).

In the second step, we searched for communities in which all associations were named by at least four participants to see even more stable structures and whether they differ from those named by at least two respondents. With this setting, the algorithm found 164 communities containing 2801 different words with community sizes between 2 and 114 members, as in [52].

**Fig 3:**
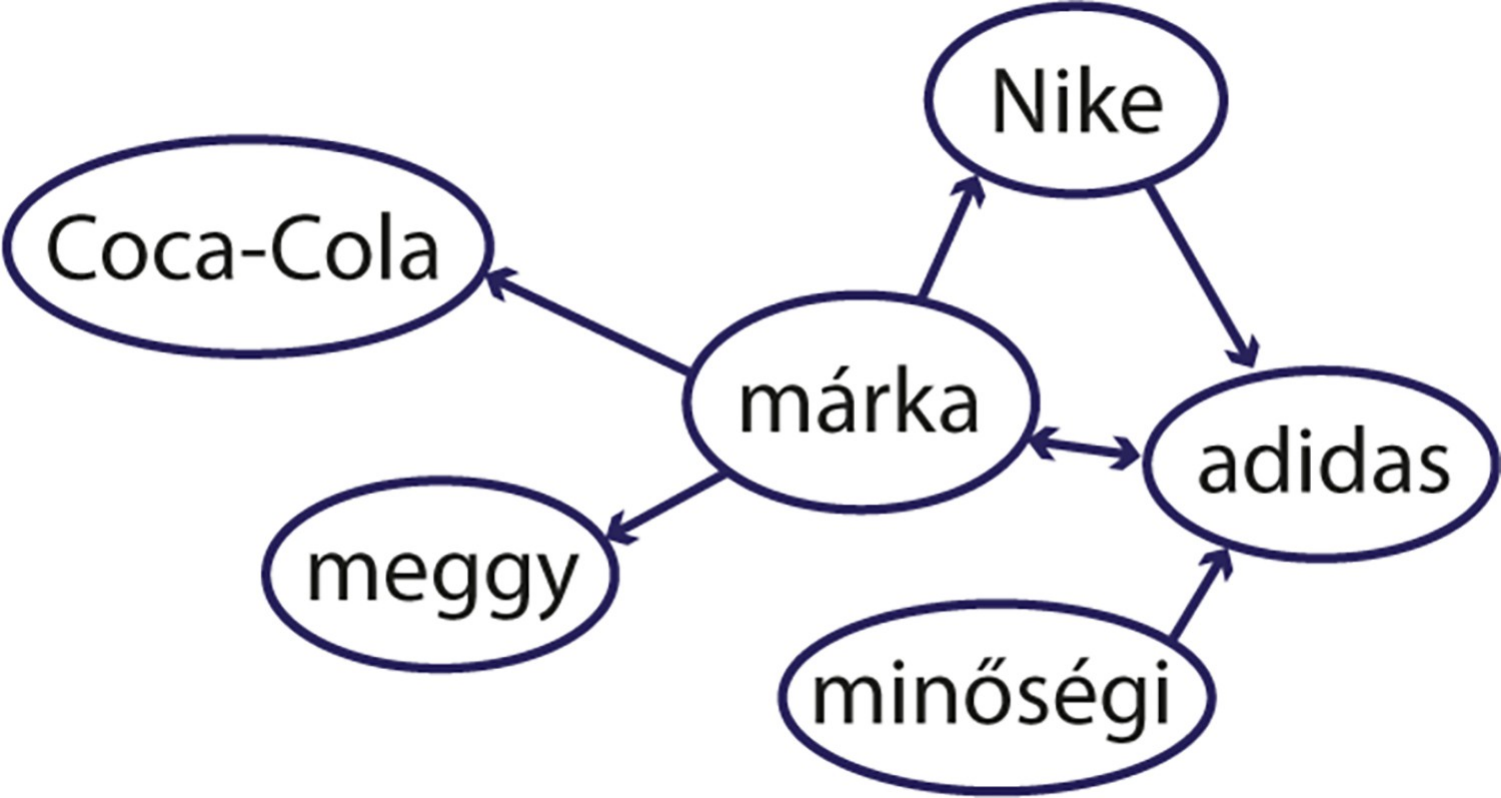
Community around the Hungarian word *m´arka*. The Hungarian word *m´arka* has a double meaning. On one side, *m´arka* means ‘brand’ in Hungarian, with the associations *Coca-Cola*, *Nike* and *adidas*. On the other side, Ma´rka is a brand name of a soft drink in Hungary, with the association *meggy* = ‘sour cherry’; a flavor of the soft drink. *Min˝os´egi* = ‘of good quality’.

Of the 164 communities, 23 contained brand names. The brand names in the communities are, in most cases, from the same product category, except community nr. 20 (Fig 3).

Of the identified communities, the community ‘news’ contains brand names of newspapers (56 words, 10 brand names), the community ‘shopping’ has supermarkets and stores (63 words, 11 brand names), and in the community ‘computer’, we found the names of services like *Google* and manufacturers like *Dell* (80 words, 8 brand names).

The community ‘traffic’ (86 words) has 20 brand names, cars and motorcycles: *Astra*, 327 *Audi*, *Benz*, *BMW*, *Ducati*, *Ferrari*, *Ford*, *Harley*, *Honda*, *Kawasaki*, *Mercedes*, *Opel*, 328 *Simson*, *Skoda*, *Suzuki*, *Toyota*, *Trabant*, *Yamaha*, *Volkswagen*, *Volvo*.

According to our findings, the overlapping and non-overlapping methods provide a different perspective on the community structure of the examined word association network. The hub percolation and LEMON methods aim to provide dense, highly overlapping community structures, resulting in a high number of communities, a considerable amount of overlaps between them, and a large number of brand names inside them. This allows us to examine the relationships between multiple brands from multiple product categories at the same time, giving us an overall picture of how brands in different product categories interact in a consumer’s mind.

In contrast, the community structure discovered by the modularity maximization method, a traditional non-overlapping approach, is more homogeneous and focused on individual brand names from the same product category. It also shows, in some cases, which product categories are related to each other in some way. This enables us to discover the associative structures consumers have around a product category in their mind, including brand names, product and brand properties, and perceived benefits and contexts of brand and product use. Some of the properties of the community structures found by the used methods and their configurations can be found in Table 1.

### Example: Colors attached to brands

For the heterogeneous community detected by the modularity maximization method presented earlier–*Ferrari*, *Greenpace*, *Fradi* (Hungarian soccer team), *Jonathan* (apple) and *Snow White* and the *Smurfs–*we could not find a single organizing principle or topic. We found, however, that in this community, colors and color names were partly dominant; besides the color names *blue*, *orange*, *green*, *black*, *pink*, *rosa*, *yellow*, *azure*, *red* in two forms, *‘vo¨ro¨s’* and *‘piros’* (cf. Benczes and To´th-Czifra [104]), the words *color* (both in Hungarian and English) and *multicolored* appeared. The color *white*, however, is connected to the community ‘health/hospital’.

The heterogeneous community also contains 11 fruit and vegetable names and names of trees. The appearance of the above-mentioned brand names in this community could be connected to their distinctive colors: *Greenpace* has green in its name, and the *Fradi* (Ferencv´aros) soccer club is often referred to as the green-whites in Hungary because of their distinctive club colors. The relatedness to color for *Snow White* and the *Smurfs* is evident. *Jonathan–*apple name–can be explained both by its color and that it is a fruit. The only remaining brand is *Ferrari*. In the case of *Ferrari*, the connection to the color red is part of the brand’s marketing strategy [105]. The strong connectedness of the brand to the color red was also shown in a different association experiment, where the examined car brands all evoked the association *car* in the first place, with the exception of *Ferrari*, where the strongest association was *red* [106].

We found that this heterogeneous community is organized around two main topics, ‘colors and fruits’, and the appearance of brand names is due to the strong connections between the brand and a distinctive color.

## Discussion

### Brands as parts of communities

As results obtained using different algorithms show, brand names are part of communities in the mental lexicon (RQ1). Although the result is not surprising, it shows that brand names are integrated into the mental lexicon, and they build relationships and are part of communities like ‘normal’ words. It is also notable that–again independent of the method used–brands are seldom standing alone. When a community has a ‘brand member’, it is likely to have another one (or several ones) also from the same or a related product category. This could be due to and explained by the organization of the lexicon, which corresponds to the first law of association: ‘associations between ideas are based on contiguity, similarity or contrast’ ([25]: 3). However expected the results are, to the best of our knowledge, there has been no empirical evidence for communities containing brand names in the mental lexicon.

### Overlapping and non-overlapping communities

This leads us to the second research question (RQ2): Which method (overlapping vs. non-overlapping) is better for community detection?

Based on the results, both methods and all algorithms presented valuable insights.

1. By using non-overlapping communities, we obtain data that is easy to analyze and draws a clear picture of communities. Every brand is part of only one community, and the connections are easily analyzed and implications easily understood. In favor of non-overlapping communities are the relatively easily manageable results.
2. On the other side, the mental lexicon is a construct that overlaps in a natural way; every word can be used in different contexts and considered the lexicon as a multilayer network, an overlapping character is inevitable (cf. [50, 52]). Using overlapping methods, however, we face (even in the current paper) the problem that too many communities are identified and differentiation between them is not easy. We would welcome a result where a brand (e.g. Ferrari) appears in two communities and two contexts: cars (as a product category) and color (as a distinctive brand characteristic). This would be a result that could be expected; as seen in the literature review, product meaning and brand meaning are interacting entities.

Running the LEMON algorithm, however, we found that 18 communities contain the brand name Ferrari (and 28 communities contain Volkswagen). It is clear that 18 (or 28) meanings of a brand do not exist. For Ferrari, we could name as broad categories 1) the brand context (color, logo), 2) the product context–and/or 2a) sportscar and/or 2b) luxury car, 3) another product context for luxury products and, perhaps, 4) a lifestyle context together with brand use. Seeing the communities of LEMON, however, we found that these possible contexts are simultaneously present in each community.

The overlapping community detection method provided good results as it managed to identify communities and brands in the communities, and the communities themselves are explainable in the product-brand context. However, the method failed to distinguish between brand and product meaning.

We are aware that current results would suggest that better–marketing-relevant–results can be obtained by using only non-overlapping methods for community detection. It must be stressed, however, that the structure of the mental lexicon is overlapping by its nature; therefore, community detection methods must be improved to provide results that can also be used for practical purposes (e.g. showing the exact contexts brands are part of in our minds).

### Brand associations vs. associations around brands

Our results show that we must distinguish between brand associations and associations around brands (RQ3).

Brand associations are the associations brands trigger. However, as we have seen in the case of communities, associations do not have to be connected to a brand name to be in the same community as the brand name (cf. point 3). This result aligns with secondary and tertiary associations described by Keller [7]. Secondary and tertiary associations are not connected to the brand itself but to an association that is connected to the brand (cf. indirect associations by Franzen and Bouwman [6]). We argue, however, that the degree of the association (secondary or tertiary) is not important, but the role and position of the association in the associative community around brands.

The question arises: Why are community detection methods better as simple associations to explain the mental position of brands (RQ3)?

**Fig 4:**
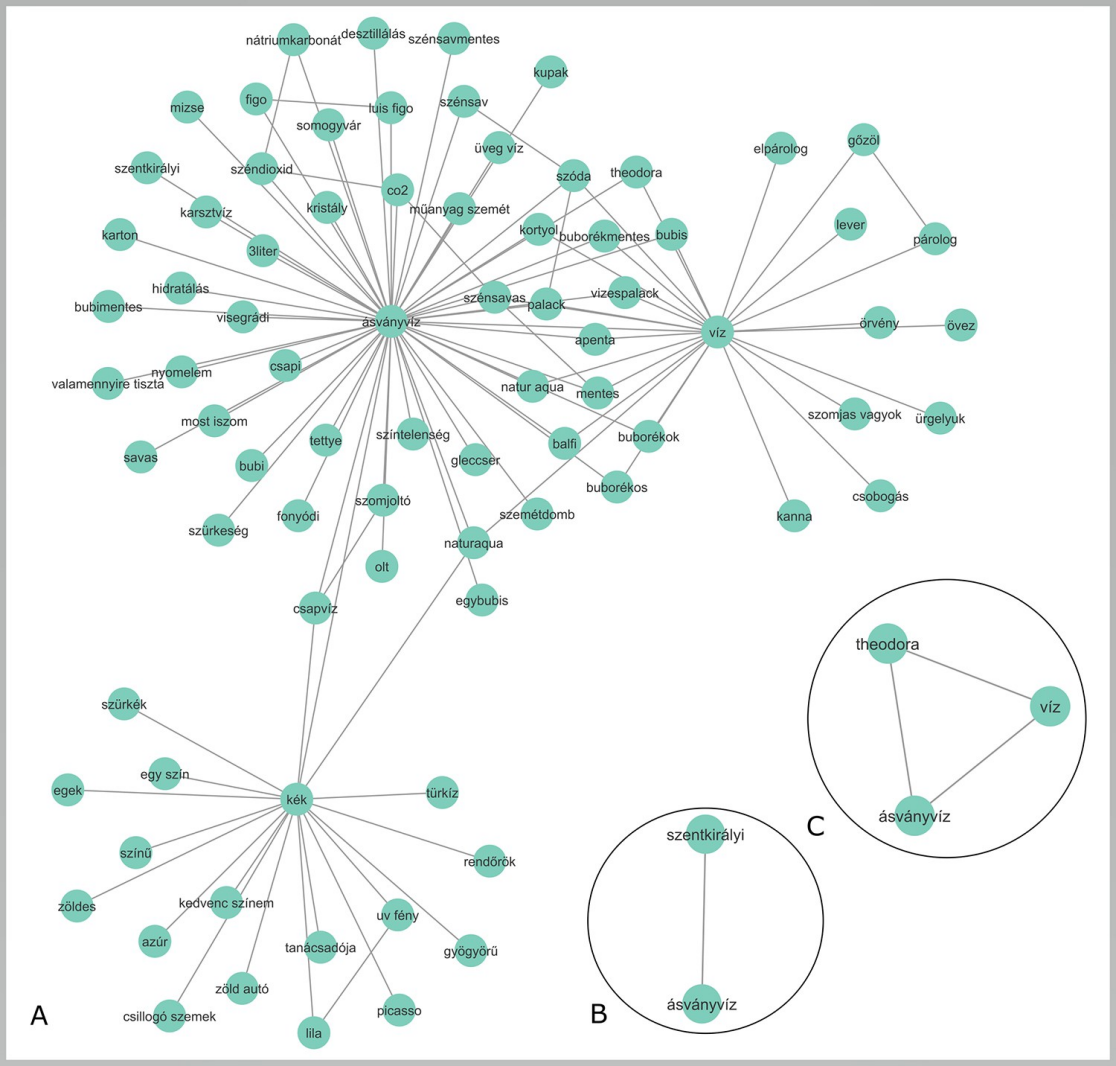
a) The community around mineral waters with LEMON (containing 7 mineral water brands); b), c) the direct connections of the mineral water brands Theodora and Szentkirályi in the database (ásványvíz = mineral water, víz = water).

**Fig 5:**
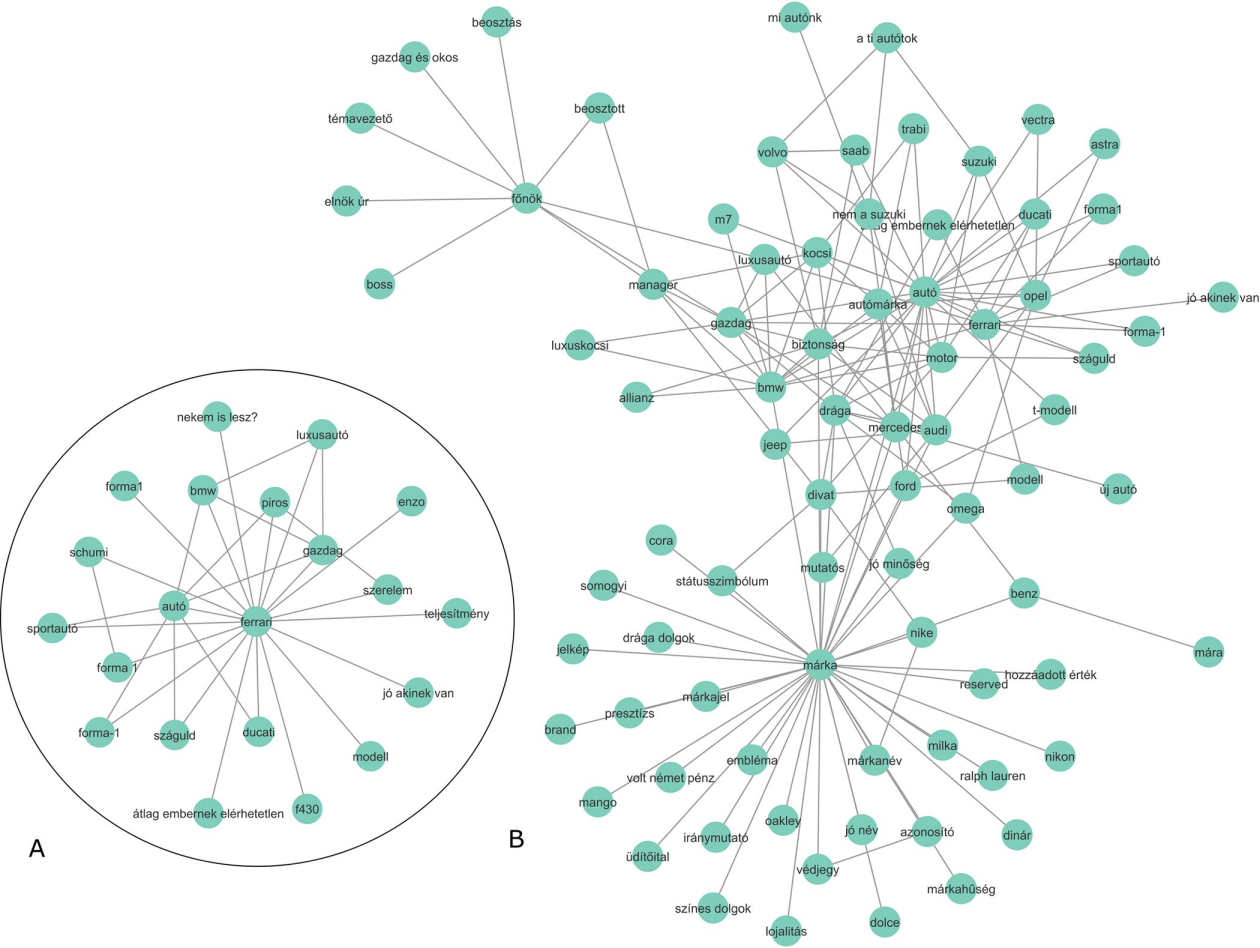
a) The direct connections of Ferrari; b) the community around Ferrari with LEMON.

To answer this question, we must first consider the above description of secondary and tertiary associations; these are the associations that are not directly connected to the brand. How can we trigger these associations? Clearly, naming the brand as a cue word is not enough as it collects only the brand’s direct associations. We can also come up with some ideas about which words would trigger the brand name itself (e.g. the product category or some distinctive brand properties, like the color of Ferrari), but it is unlikely we will test all possible triggers. Here word association data could come into play; they are collected not focused on a given brand but focused on the research of the mental lexicon. Therefore–as we have seen–brand names will be part of these databases, but they are not the focus. As an example, we take two Hungarian mineral water brands, Theodora and Szentkirályi (Fig 4A–4C), and Ferrari (Fig 5A and 5B).

As we can see, the direct network is not complex, while the community structures present a more complex structure with secondary and tertiary associations, showing not only the structure in the immediate vicinity of the brand.

Brand association data is collected by presenting the brand name as a stimulus, thus picking out an entity (a brand) from the complex networked structure of the mind and analyzing the picked out entity alone, without knowing the surroundings it naturally has in the consumer’s mind. This shortage of brand analysis is known to research; hence, the recommendation to also study secondary and tertiary associations (cf. Keller [7] or Franzen and Bouwman [6]).

To compare a brand’s associations to those of other brands, and to see similarities and differences, it is important to analyze the complex structure the brand is part of; at this point, the identification of communities in the mental lexicon comes into play.

The information stored in the communities containing brand names fell, in most cases, into the following categories (examples drawn from the community ‘cars’; modularity maximization nr. 2, 86 words):

- brand names (Audi)
- brand/product characteristics (speed, noisy, PS)
- brand/product parts and ingredients (wheel)
- contexts where the brand/product is used (traveling, race)
- results of using the brand/product (accident, speeding)
- substitute product (bicycle)

The observed words in the identified communities are in line with brand associations in general [1, 6, 7]. Our results verified the results of Aaker [1], Franzen and Bouwman [6] and Keller [7]–it is important to note, however, that 1) in our research, word association data was the corpus of research, where brand names just appeared in the database, and 2) we did not analyze which associations are connected to the brand name itself; we just identified communities where brand names were present.

Although the starting point of our research was not brand names–we did not initially use brand names as cue words–we identified associations that are in line with brand-focused research. These results show that when describing a brand’s cognitive position, it is advisable to take into account not only the associations a brand is directly connected to but also the complex associative structure around brands.

In this regard, the communities contain brand information and product information (see previous point and literature review). We surmise that the associations of brands define the meaning of the brand, while communities around several brands define the meaning of a product category. Thus, communities do not necessarily provide relevantly more information about a brand compared to other marketing research methods; rather, word association data and communities can help to gain a deep insight into product category information and the interacting connection between product categories and brands. The method has the advantage of using one large dataset to answer several product- and brand-related research questions with the same dataset and directly compare the differences and similarities of different brands (thank you for a reviewer to pointing out this advantage).

In addition, the gained information may help to plan advertisement campaigns more precisely; words from the community (which are not directly connected to the brand) may help to formulate messages, which are more easily accepted by the target group since the words are already part of the community. However, at the same time, words missing from the communities can also show what words may be used for differentiation from other brands (see Implications).

### Brand names as part of the encyclopedic knowledge layer

At this point, it is important to emphasize an assumption that arises from previous research but has to be verified empirically in the future.

As described in 2.1, lexical units store several different pieces of information in the mind, and the stored information enables an effective organization of the units and helps to connect them [21, 107]. Existing research shows that the units are parts of a network in the mind, more precisely, multiple layers of networks [108]. Existing research also assumes that a layer storing encyclopedic knowledge exists in the mind [33, 52].

Since brand names were present in word association experiments, we assume that brand names are represented as nodes in the mind, connected to other words and brand names (cf. Esch [67]; Franzen and Bouwman [6]).

**Fig 6:**
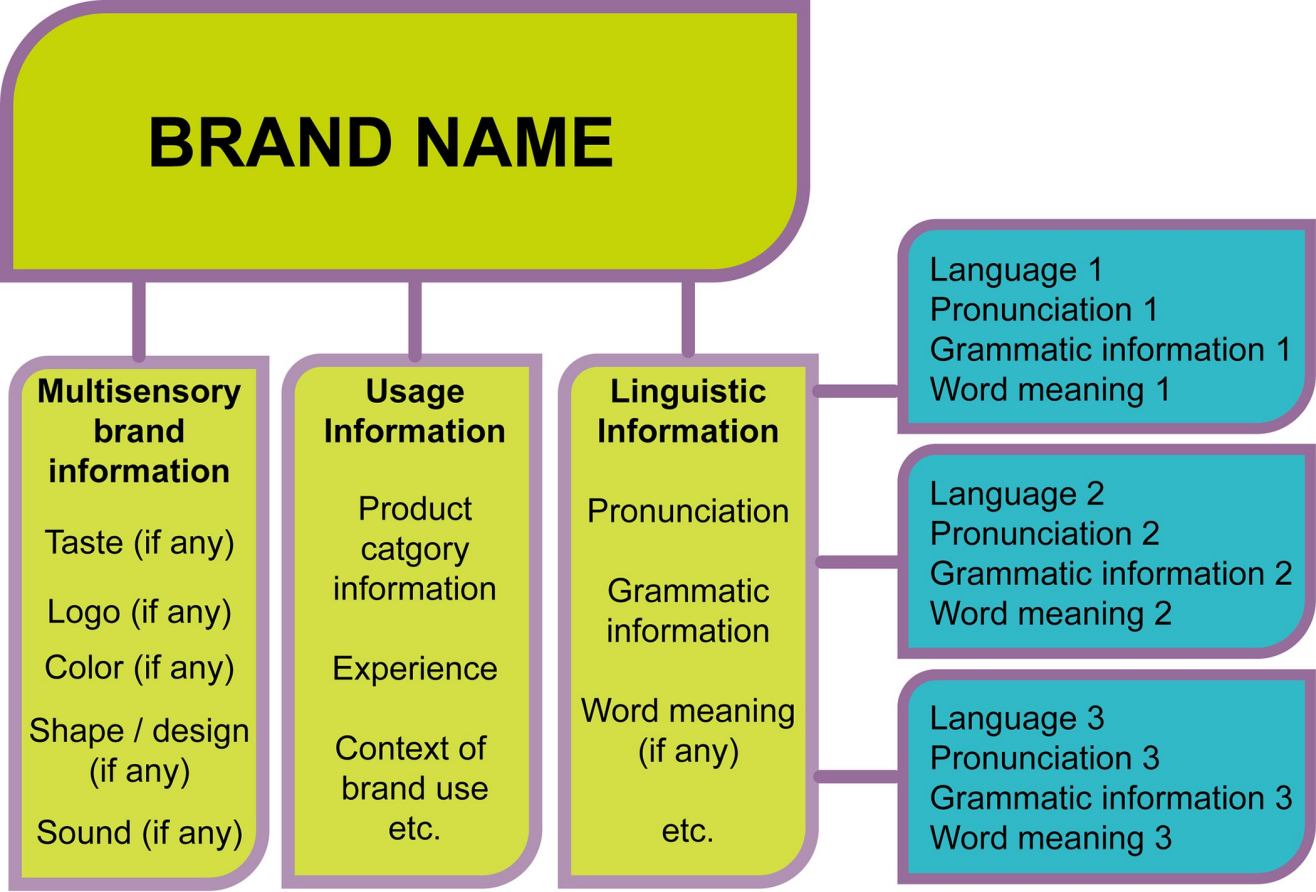
Brands as part of the encyclopedic layer.

From our results and from research on the layers of the lexicon [52], we propose that brand names are part of the encyclopedic layer in the mind (Fig 6), which is not language-specific–meaning that, for example, *Coca-Cola* is not stored as a specific Hungarian or Croatian or German word, but as a unit that can be connected to several languages at the same time.

This implies that the information stored at this node in the mind is not exclusively language-specific; we store certain multisensory and usage-specific knowledge of the brand and the product (e.g. taste, color of the packaging, shape of the logo) and some language-specific information (e.g. pronunciation of the brand name, slogan, advertisement text). Pieces of this multisensory information are always present and active, regardless of the language and linguistic context. For example, driving in a foreign country and seeing the logo of a fast-food brand, all the information about the brand, products, price category and tastes will be activated, even if one does not speak the language of the given country.

This assumption–if verified–could indicate that for a global brand, it is important to use brand communication patterns, which are not country- and language-specific, meaning that, for example, texts should not be the focus of an advertisement. The focus should be multisensory, language-independent information, which in the consumer’s mind does not need to be connected to a given language, thus enhancing the storage of brand-related information on the encyclopedic layer. This may result in brands relying on techniques that are accessed more easily in a context where text is less relevant (e.g. in a foreign country).

Several well-known global brands seem to use this technique already. It should be interesting to learn whether the global success of these brands was connected to the fact that they–unconsciously to the layers of the lexicon–used the method described above in their campaigns.

We must note that, at this point, this is only an assumption based on the analysis of one database.

## Implications

### Differentiating

The description of brand names as nodes belonging to closely-knit communities in the mind shows that brand communication of a well-known or young brand cannot exist in an empty space–all the other brands and the product category the brand belongs to and the attitudes and expectations of consumers towards the product category shape how the brand is perceived.

Product-related communities–and words in these communities–may be used by new brands in a product category. First, new brands (and brand names) have to be connected to the product category in as many ways as possible to ensure that the new brand is immediately perceived as part of the given product category and thereby benefits from all the associations of the product category. This can be done by using words in advertisements, which are part of the community of the product category.

At the same time, however, they have to ensure that the community around the brand is different from the communities of all other brands and that it contains not only product-related characteristics but also a distinctive logo and slogan that are specific to the brand. A good example is the Hungarian herbal liqueur Zwack Unicum. It is a widely known Hungarian brand with over 100 years of history. It has a distinctive product feature: its bottle has a unique round shape that characterizes the brand. A previous experiment showed that the shape of the bottle frequently appears as a brand association with words like bottle shape, small round bottle, small spherical bottle, round bottle, round, shaped bottle, round, sphere [74].

**Fig 7:**
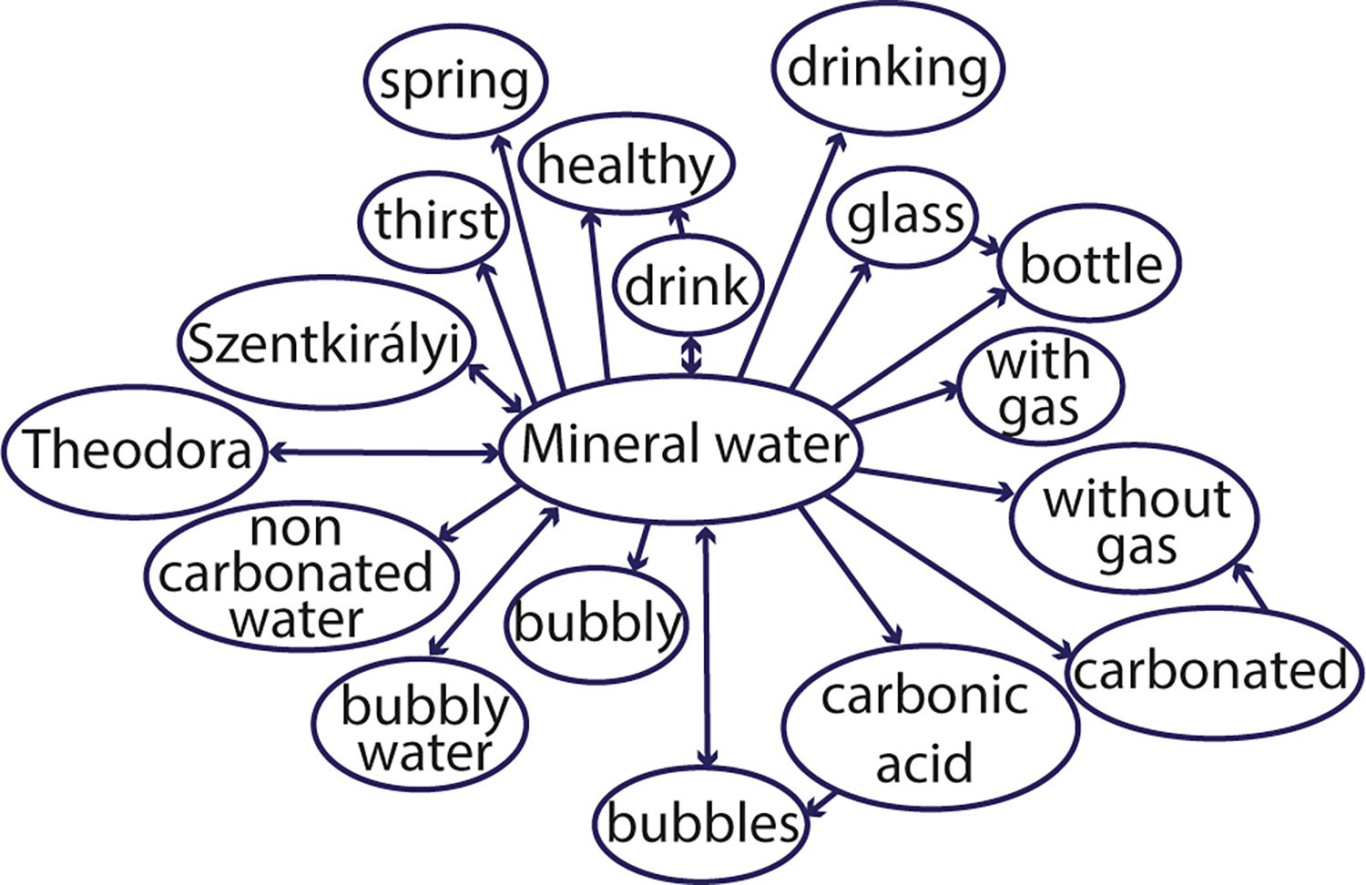
A community centered around the concept of ‘mineral water’.

Advertisements could profit from a better understanding of communities around brands [69]. An example of this can be seen in Fig 7, with a community centered around mineral water (obtained with modularity maximization, at least four respondents named the associations).

As we can see in the community in Fig 7, most words describe the characteristics of mineral waters. For a marketer, however, the word healthy should be the most important; it shows that mineral waters are generally thought to be healthy. This knowledge could help advertise a new brand, emphasizing and underlying its healthiness–and in advertisements, the word healthy should appear.

Why is this important? As seen, the community of the product category mineral water already contains the word healthy. Using this word in an advertisement will not help to differentiate a brand–it emphasizes, however, one important feature of the product. Since the word healthy is already part of the community centered around mineral waters, this strong connection can be used in advertising (cf. Shamsollahi [109]). On the other hand, by using the communities of associations, it is possible to connect unique (brand-specific) attributes to brands [69]. In the case of mineral waters, this attribute cannot be just healthiness–since all mineral waters are believed to be healthy–but some specific information. Advertising could use specific brand characteristics (e.g. the ingredients of the given mineral water) to emphasize why it differs (e.g. is healthier) from other mineral waters.

### Negotiated meaning

The communities around brand names are not necessarily the same as those communicated through advertisements and marketing efforts. The meaning of a brand and its perceived and accepted characteristics always develop through a negotiation process between the brand owner and society. The result of this process is the actual meaning of the brand in a given society, which can be different from the meanings the brand owner intended to engrave into the brand [85, 110].

The communities around brands and product categories can contribute to finding these negotiated meanings of brands. Neglecting these meanings can result in brand disasters. In this regard, the communities identified by our analysis can be used to see whether the brand is positioned in the mind of the consumer the way the brand owner intended. Therefore, it is important to analyze not only associations connected to a brand but also the complex associative structures and communities around the brand and the whole product category.

## Limitations

Current results are obtained from a word association database collected for linguistic research. Although some demographic variables were collected, no information was asked from the respondents about their brand or product preferences. Therefore, whether the found community structures are general or user-group specific cannot be analyzed.

It must be assumed, however, according to Franzen and Bouwman [6] (cf. [111]), that different community structures of the brand can exist in the minds of different consumer groups. We assume that current results show and detect common communities around the brand, which may differ from the community structures of ‘experts’ (e.g. brand fan communities).

## Conclusion

In this paper, we analyzed communities in a word association database and searched for brand names inside the communities.

We started with hub percolation, and results showed that the communities we detected identified communities around brands, with the communities being partly homogenous. The LEMON algorithm provided good results since it takes initially known members of communities as an input. We are interested in communities organized around previously known brand names, and we could simply provide a list of brand names to the algorithm as input, which would then grow communities around them. The communities provided by the modularity maximization algorithm were homogeneous and focused on individual brand names from the same product category, enabling us to better explore the associative structures around product categories.

Our results showed that brand names are present in word association databases, and we found that the algorithms were able to find homogeneous communities in these databases. We also showed that communities contain brand names from the same product category since brands of a given product category share common characteristics.

Based on the results presented here and our previous works, we described how brand names are stored in the mind; they are multisensory entities strongly connected to the product category. We also showed that overlapping and non-overlapping algorithms both provide valuable insights, but currently, non-overlapping methods provide results that are easier to interpret. We also showed that communities in the mind play several roles; communities around brands define the meaning of the brand, while communities around several brands of the same product category define the meaning of a product category.

Future research has to describe a brands’ position in these communities more precisely. This can happen by analyzing larger association databases or through target-specific large-scale data collection in several languages. In further research, the characteristics of the test participants must be taken into account, for example, their gender, age or consumer habits. A better understanding of communities in the mind contributes to the understanding of brands as psychological constructs, enabling the use of more effective brand management practices in the future.
